# Primary Involvement of Allografted liver in Post-Transplant Lymphoproliferative Disorders, Report of Two Pediatric Cases and Review of the Literature

**DOI:** 10.5812/ircmj.1134

**Published:** 2012-11-15

**Authors:** Bita Geramizadeh, Sama Nikeghbalian, Seyed Mohsen Dehghani, Ali Bahador, Heshmatollah Salahi, Seyedali Malekhosseini

**Affiliations:** 1 Department of Pathology, Shiraz University of Medical Sciences, Shiraz, Iran; 2 Department of Hepatobiliary Surgery, Transplant Research Center, Shiraz University of Medical Sciences, Shiraz, Iran; 3 Department of Pediatrics, Shiraz University of Medical Sciences, Shiraz, Iran; 4 Department of Pediatric Surgery, Transplant Research Center, Shiraz University of Medical Sciences, Shiraz, Iran; 5 Department of Surgery, Transplant Research Center, Shiraz University of Medical Sciences, Shiraz, Iran

**Keywords:** Liver, Lymphoproliferative Disorders

## Abstract

Post-transplant lymphoproliferative disorder is a lymphocyte proliferating disease, usually of B cell origin, and rarely of T cell. Involvement of liver itself in liver transplant recipients as the primary organ is not common. Herein we report our experience in two patients who primarily presented in the allografted liver, both of whom were promptly diagnosed after liver biopsy and treated successfully .Now after a few months; both of the patients are alive with normal liver function tests and negative imaging studies.

## 1. Introduction

Post-Transplant lymphoproliferative disorder (PTLD) consists of a heterogeneous group of lymphoproliferative disorders which occurs in.1-4.7% of orthotopic liver transplantations (OLT) and its incidence depends on the age of patient and immunosuppressive therapy used (1). PTLD in OLT patients typically presents as a systemic illness and it rarely presents with involvement of hepatic allograft (2). Among more than 1000 OLT patients in our center we had two patients presented with PTLD in the allografted liver. Herein we report our experience with these two liver transplant patients who had biopsy proven liver involvement.

## 2. Case Presentation

### 2.1. Case 1

A 3-year-old boy underwent OLT for cirrhosis secondary to tyrosinemia (HBS and HCV negative). His immediate post-transplant course was unremarkable. After 6 months he developed constipation, vomiting and fever (T> 38о C). At that time he was receiving Tacrolimus, mycophenolate mofetil and prednisolone. His laboratory findings are summarized in Table 1.

**Table 1:** Laboratory findings in 2 patients with PTLD presentation in the allografted liver

	Case No-1	Case No-2
**ALT ^a^**	75 IU/L	69 IU/L
**AST ^a^**	34 IU/L	266 IU/L
**Alk-P ^a^**	371 IU/L	200 IU/L
**LDH ^a^**	530 IU/L	450 IU/L
**CRP ^a^**	positive	positive
**EBV-VCA ^a^**	positive	positive
**WBC ^a^**	3200/ml	3700/ml
**Hemoglobin**	8.2gr/l	7.2 gr/l
**Platelet**	100000/ml	120000/ml

^a^ALT: Alanine aminotransferase (normal<41IU/L), AST: Aspartate aminotransferase (normal<37IU/L), Alk-P: alkaline phosphatase< 1200 IU/L), LDH: lactate dehydrogenase (normal<500 IU/L), CRP: C-reactive protein, EBV-VCA: Epstein Barr-virus viral capsid antibody, WBC: White blood cell count

**Figure 1:**
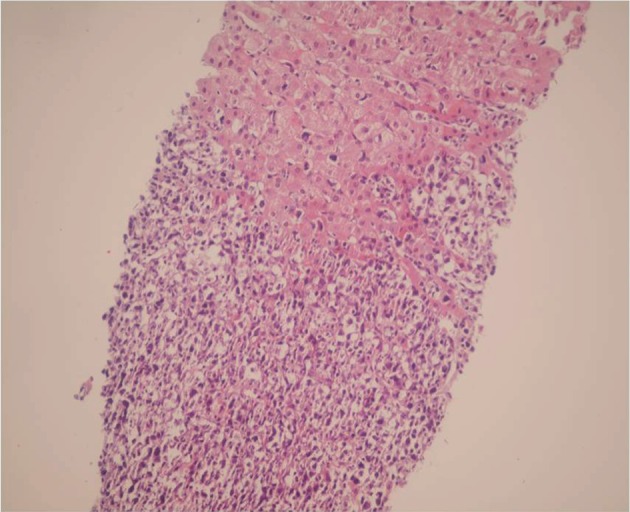
Liver Biopsy Shows Infiltration of Lymphoma Cells in Two Cases. (H&E X 250)

**Figure 2:**
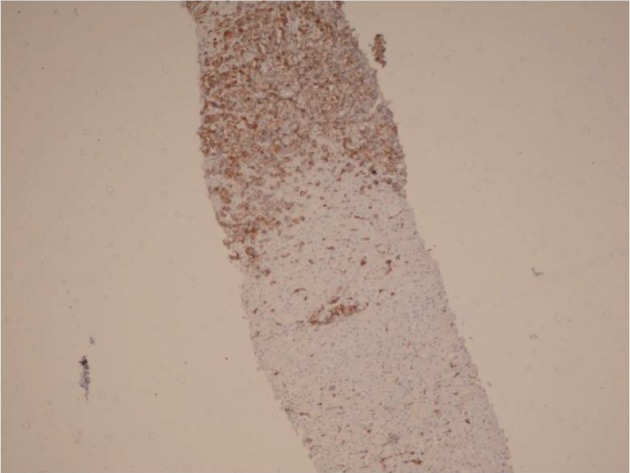
Immunohistochemistry Shows Reactive Lymphoma Cells with CD 20

Liver ultrasonography showed a hypoechoic lesion measuring 14x16 mm in medial segment of left lobe. Liver biopsy was performed which showed severe infiltration of highly atypical mononuclear cells in the portal tracts with parenchymal involvement ( Figure 1 ). All of these cells were reactive with B cell markers such as CD 20 and CD 79 ( Figure 2 ). LMP-1 antigen of EBV was positive in some of the above mentioned atypical cells. EBV-PCR was also positive in the liver tissue.

With the diagnosis of monomorphic PTLD (B cell type), doses of immunosuppressive drugs were reduced, but he remained febrile and gradually became severely pancytopenic. Bone marrow aspiration and biopsy were performed which showed lymphomatous involvement. Standard chemotherapy of CHOP regimen (cyclophosphamide, adriamycin, vincristine, and prednisolone) plus Retuximub was started for him .Now after 9 months, he is doing well and in an acceptable condition. There is no evidence of tumor recurrence in both imaging studies and laboratory tests.

### 2.2. Case 2

A 16-month-old male underwent OLT for Crigler-Najjar syndrome (HBS and HCV negative). He had an uncomplicated post-transplant period on Tacrolimus, mycophenolate mofetil and prednisolone. After 6 months he developed fever and abnormal liver function tests. His laboratory data are shown in table-1. Imaging studies didn't show any focal lesion. Liver biopsy was done which showed portal infiltration of atypical lymphoid cells with irregular nuclear border (Fig-1b) which were positive with CD 20 and negative with CD3, and CD30.Although LMP-1 by immunohistochemistry was negative but PCR for EBV was positive in the liver tissue. The immunosuppressive drugs were reduced, but he remained febrile and after about 2 weeks he developed cervical lymphadenopathy, lymph node biopsy showed diffuse large B cell lymphoma, so CHOP regimen plus Retuximab was started. After a couple of weeks, the enlarged lymph nodes were disappeared and now after 6 months, he is alive and symptom-free. Imaging studies including ultrasonography failed to show significant finding, and imaging of the transplanted liver is normal.

## 3. Discussion

PTLD is a potential complication of transplanted patients with varying incidence based on the transplanted organ and immunosuppressive regimen (3). PTLD in OLT patients typically presents as a systemic illness with rare presentation in the hepatic graft (4). PTLD in the allografted liver as a part of systemic disease or alone has been reported from 0 to 8% in pediatric group from different centers (5-7). Therefore, it seems that liver localized PTLD as the first presentation in pediatric age group is rare (2).

There are rare reports concerning the differences between PTLD presenting in the liver allograft and systemic PTLD. Capello et al (8) in their report have described this issue and concluded that liver allograft PTLDs are of donor origin, mostly B cell type, early onset and pathogenetically related to EBV infection as our two patients. In an OLT patient, PTLD with liver involvement presents with variable symptoms such as fever, chills, nausea and jaundice (9). Both of our patients presented with fever and abnormal liver function tests and liver biopsy was performed to exclude a rejection episode and to diagnose the source of fever. EBV plays a special role in the pathogenesis of lymphoproliferative disorders that develop in the grafted liver (9, 10). EBV has the ability to stimulate B cells to proliferate and transform. After primary infection, EBV persists in the B cells and causes a latent infection. In the process of latency during reactivation after immunosuppression, it can cause lymphoproliferative diseases such as PTLD (11). In both of our cases association with the EBV was definite, because the EBV-PCR in the liver tissue was positive. Some authors believe that the type of immunosuppressive regimen play a crucial role in the pathogenesis of PTLD specially OKT3, antilymphocyte globulin (ALG) and antithymocyte globulin (ATG), but others found no significant association except for treatment with antithymocyte globulin (1, 12).

The first step of the treatment in PTLD is decreasing immunosuppression (11). In none of our patients, this was curative and both of them required chemotherapeutic regimen including Retuximab and standard protocol. After that a clear regression of liver lesions was noticed and liver function tests became normal. According to our experience, the liver localization of PTLD does not change the management of PTLD, i.e. chemotherapy and decreasing immunosuppression can induce subsequent remission without graft rejection. This finding has also been reported in previous studies (12, 13). Our experience in these two patients insists on the value of the early diagnosis of PTLD by performing liver biopsy, so clinicians taking care of the OLT patients should consider PTLD with liver involvement or liver localized PTLD as the possible cause of abnormal liver function test and fever (14, 15).
